# Factors Affecting Vagus Nerve Stimulation Outcomes in Epilepsy

**DOI:** 10.1155/2021/9927311

**Published:** 2021-08-03

**Authors:** Mehdi Abbasi, Atie Moghtadaie, Seyed Amir Miratashi Yazdi

**Affiliations:** ^1^Radiology Department, Mayo Clinic, Rochester, MN, USA; ^2^Internal Medicine Department, Sina Hospital, Tehran University of Medical Sciences, Tehran, Iran; ^3^General Surgery Department, Sina Hospital, Tehran University of Medical Sciences, Tehran, Iran

## Abstract

Epilepsy as a common neurological disease is mostly managed effectively with antiepileptic medications. One-third of patients do not respond to medical treatments requiring alternative therapies. Vagus nerve stimulation (VNS) has been used in the last decades for the treatment of medically resistant epilepsy. Despite the extensive use of VNS in these patients, factors associated with clinical outcomes of VNS remain to be elucidated. In this study, we evaluated factors affecting VNS outcomes in epileptic patients to have a better understanding of patients who are better candidates for VNS therapy. Several databases including PubMed, Scopus, and Google Scholar were searched through June 2020 for relevant articles. The following factors were assessed in this review: previous surgical history, age at implantation and gender, types of epilepsy, duration of epilepsy, age at epilepsy onset, frequency of attacks, antiepileptic drugs, VNS parameters, EEG findings, MRI findings, and biomarkers. Literature data show that nonresponder rates range between 25% and 65%. Given the complexity and diversity of factors associated with response to VNS, more clinical studies are needed to establish better paradigm for selection of patients for VNS therapy.

## 1. Introduction

Epilepsy is one of the most prevalent neurological conditions involving nearly one percent of the world population. Despite optimal and successful management of epileptic patients with appropriate antiepileptic drugs, 10–30% of patients do not respond well to these treatments and are categorized as medically resistant epileptic patients [1, 2].

The quest for identification of treatments for neurologic disorders and brain abnormalities have led to the development of a treatment method called vagus nerve stimulation (VNS). Historically, the inhibition of motor activity by stimulation of vagal afferents was first reported in 1937 by Schweitzer and Wright [3]. VNS which consists of chronic, intermittent stimulation of vagus nerve (usually left side) has been approved by FDA [1, 3, 4] for treatment of partial-onset, drug-resistant epileptic patients who are not suitable candidates for curative surgical resection or patients in whom resective surgery has provided no benefit. Since the early 1990s, VNS has been used as an alternative treatment in medically resistant partial epilepsy [5]. Being initially approved for patients with 12 years of age and older, investigations of recent decades have shown that VNS is a safe and effective method for adults and children of all ages suffering from different types of epileptic disorders [6, 7]. A cohort study evaluated VNS for more than 10 years and concluded that VNS can decrease the seizure frequency and seizure intensity and enhance the patients' mood. Low morbidity and sustained therapeutic effects of VNS have been also mentioned as advantages of this treatment. [8] Thus, uncontrolled seizures heavily impact the patients' functional neurodevelopment and subsequent quality of life. Considering the cumulative effect of VNS in reduction of seizure frequency and severity, VNS has become a valuable modern option in the therapeutic armamentarium of patients with intractable epilepsy [7]. It is well documented that, in patients who do not show reductions in seizure frequency, there are still significant improvements seen in alertness, attention, and concentration levels of patients, reduced occurance of status epilepticus and its subsequent hospitalization, or increases in seizure-free days [7]. These effects promote the level of quality of life in epileptic patients, regardless of the efficacy of VNS in reducing seizure frequency. Thus, improved behavior, language, or sleep and expression of the desire for continuation of VNS are frequently reported [6, 9].

The efficacy of VNS improves significantly with time, which supports the hypothesis of a cumulative effect [10–14]. A 17-year follow-up showed 50–90% seizure frequency reductions in 38.4%, 51.4%, 63.3%, and 77.8% of patients at 1, 2, 10, and 17 years after VNS implantation. Almost all published data analyzing the long-term outcomes of VNS reported improved outcomes over long periods of time [15, 16]. It is well established that seizure burden progressively reduces with continued use of vagus nerve stimulator [17, 18]. The exact mechanism for the improving efficacy of VNS with prolongation of therapy is not fully understood. Chronic therapeutic response to VNS therapy is highly associated with bilateral thalamic increases in synaptic activity. During chronic VNS therapy, brain excitatory amino acid neurotransmitter levels are reduced and inhibitory neurotransmitter levels are increased but no direct relationship with seizure control has been found [14]. García-Pallero et al. reported that VNS effectiveness improves over one year because of an accumulative effect on seizure [19]. Vagal nerve stimulation (VNS) response is not immediate. A progressive reduction in seizure frequency usually occurs during a period of 12–18 months after implantation [19].

Despite substantial amount of data, there is no clear evidence regarding the group of patients in which VNS would be most beneficial. Identifying prognostic factors not only increases our knowledge of mechanisms by which VNS reduces seizures but also prevents us from imposing unnecessary surgical procedures or financial burden on patients. In this review, we discussed and reviewed current literature on potential predicting factors that could determine the outcome of VNS therapy.

## 2. Methods

This narrative review on the factors affecting VNS outcomes in epilepsy includes studies in which epileptic patients of any type were treated with VNS. Comprehensive literature search in PubMed, Scopus, and Google Scholar databases was conducted through June 2020 to identify relevant articles. Keywords of “vagus nerve stimulation,” “VNS,” “epilepsy,” “seizure,” and “prognostic factors” with various combinations were used. 669 studies were found in the initial query. Then abstracts of studies were reviewed for relevance of the studies. The majority of studies were not focused on the topic of our review. References' lists of selected articles were also hand-searched for related articles. Abstracts of articles were reviewed for final inclusion of articles in the study.

## 3. Results

45 studies are reviewed in our study. The summary of studies is presented in Table 1. Detailed results are provided in the following sections.

### 3.1. Patients' Characteristics

Patients' clinical characteristics that had been suggested to predict responsiveness to VNS therapy have been proven to be elusive [33]. These factors include a wide variety of parameters including previous history, age at implantation, and gender, which are reviewed separately in the next sections.

### 3.2. Previous Surgical History

It is speculated that the structural changes consequent to surgical interventions in the brain may play some critical roles in response to treatment for epileptic patients. Labar suggested that patients who had previously undergone corpus callosotomy respond well to VNS [31]. Kawai et al. also emphasized that previous resective surgery and residual pathology do not influence the efficacy of VNS in treatment of epilepsy [10]. Although available data on the impact of previous surgical history on VNS efficacy is scarce, it can be inferred from the available studies that this factor does not significantly alter the VNS outcome.

### 3.3. Age at Implantation and Gender

There was no report on correlation of gender and clinical VNS outcome in the literature [16]. In contrast, the relationship between age at implantation and clinical response to VNS is an issue of controversy with conflicting results in the literature. [29] Although the initial FDA approval of VNS was specifically granted for age group of older than 12 years, a growing bulk of lines of evidence have strongly suggested that VNS efficacy in younger age groups is remarkable. Heterogeneous study designs, different sample sizes, and various follow-up periods in the studies make the conclusion on application of VNS in younger epileptic patients a challenge. Although precise statistical conclusion is not feasible from available studies, the general trend in the current literature favors none of the age groups over another in terms of response to VNS therapy. Hereby, we review the findings regarding the age and response to VNS in the literature.

Dede Ho et al. reported that there was no significant correlation between the age of patients, age at VNS implantation, epilepsy onset age, and VNS outcomes [25]. Chrastina et al. found that older age was not a negative predictor of VNS efficacy [23]. Several other studies also rejected the association of VNS outcomes with age at implantation, age at onset, or patients' age [6, 19, 33].

In contrast to studies denying the role of age in clinical response to VNS, there are numerous studies indicating the important role of age in achieving therapeutic goals. Ghaemi et al. reported better response in patients below 18 years of age at the time of implantation compared to those above 18 years of age [29]. This result could be explained by the fact that an immature brain has a higher threshold for excitatory stimuli and seizure-induced changes than an adult one. Another report has claimed that there is a higher percentage of responders in patients below 18 years of age at implantation of VNS. In this cluster of patients, there were no differences when classifying patients for age of onset of epilepsy and preimplant epilepsy duration. In [26], Colicchio et al. reported that the clinical outcome in preadolescent age group (0–12 years) is slightly better than that in adolescent age group (13–18 years) and much better than that in adult patients. The significant difference was between “very young” (0–6 years) and adult patients. Very young patients who had the highest averages of seizure frequency at baseline showed the highest percentages of seizure reduction [11]. Thus, significant prognostic factors for the end point of “time to the first best response” at univariate analysis were age at implant and lesional etiology [29]. These findings suggest that earlier intervention in the course of disorder may lead to improved efficacy of VNS [1]. Lagae et al. reported that the only factors which influence the outcome were age at implantation and duration of epilepsy [4]. Statistically, there was a correlation between younger age at implantation (*<*5 years compared to *>*5 years) and seizure freedom [4]. Englot et al. reported that seizure freedom occurred in only 8.2% of patients aged between 4 and 48 months [2]. Labar found greater seizure rate reductions in patients above 32 years of age compared to younger patients, whereas Wernicke et al. found greater seizure rate reductions in patients below 34 years of age [31]. Kuba et al. found lower response rates in the children compared to the whole group (the age ranged from 13 to 64 years). This finding can be attributed to the patient selection in that study with the children suffering from more severe epileptic conditions [15]. As mentioned, there is controversy going on regarding the efficacy of VNS in various age groups. Still, there is no consensus on the age cut-off for when VNS is most effective. Ages from 5 to 34 years were reported in the literature as cut-off points for comparison of age groups.

### 3.4. Types of Epilepsy

Initially FDA approved VNS for treatment of patients with partial-onset seizures but the clinical applications of this procedure have expanded over the last 20 years. To avoid unnecessary interventions in patients, which types of epilepsies respond well to VNS must be determined.

In a study on four distinct seizure types including generalized, focal, myoclonic, and atonic seizures, it was observed that generalized tonic-clonic and atonic types had significantly more favorable outcomes with VNS compared to other seizure types [7]. These findings prompt the need for classification of seizure frequency reduction results according to seizure types to achieve a more comprehensive and accurate conclusion. [7] Another study has demonstrated that seizure frequency reduction is marginally higher compared to cryptogenic cases, but the difference could not reach statistical significance (*p* > 0.05) [11].

Analysis of symptomatic epileptic conditions including cortical malformation, ischaemia, meningoencephalitis, and tuberous sclerosis has shown that cortical malformation is associated with significantly worse clinical outcomes in comparison to postinfection cases [29]. Assessment of clinical outcomes in patients with severe multifocal epilepsy, partial epilepsy, and Lennox-Gastaut syndrome revealed that the best and worst outcomes were seen in severe multifocal epilepsy and Lennox-Gastaut groups, respectively [11]. There are also reports revealing that VNS therapy is particularly effective in patients with Lennox-Gastaut syndrome [33].

Another comparison among nonidiopathic partial epilepsy, nonidiopathic generalized epilepsy, and idiopathic generalized epilepsy demonstrated that VNS is significantly more efficacious in nonidiopathic partial epilepsy patients compared to other two groups (*p* < 0.01) [6]. Patients suffering from status epilepticus also benefit from VNS by decreasing the frequency of attack episodes [6]. As Englot et al. have stated, the response to VNS in patients with predominantly partial seizures, particularly simple-partial seizures, and auras is most favorable, while the worst outcomes are seen with generalized tonic-clonic seizures [1].

The origin and nature of seizures can also be important factors in determining the outcomes of VNS therapy. Patients with the etiology of neuronal migration disorders seem to respond less to VNS [12]. Frontal lobe epilepsy shows better response to VNS than epilepsy arising from temporal regions [5]. In contrast, Ghaemi et al. reported that VNS efficacy in patients with an onset of seizure activity in the temporal area is better than that in those with frontal or frontocentral seizure activity [29]. One study claimed that response to VNS in patients with independent seizure foci in both hemispheres may not be satisfactory [33].

There are conflicting reports on seizure frequency reduction in patients with focal epilepsy and generalized epilepsy. Rychlicki et al. [30] found that partial epilepsy had better prognosis for seizure control. Englot et al. [2] predicted a better result in generalized epilepsy. Rice and Valeriano [34] found a notable reduction in seizure frequency in those patients who had just one type of seizure compared to patients harboring multifocal seizures [18].

Lagae et al. confirmed that there was no significant difference in VNS outcome between generalized and focal epilepsies [4]. A study by Gurbani S et al. indicated that VNS therapy was successful in focal epilepsy and some types of generalized epilepsy. VNS results have been reported to be satisfactory in patients with Lennox-Gastaut syndrome and tuberous sclerosis complex. VNS was useful in achieving ≥50% reduction in seizure frequency for patients with Lennox-Gastaut syndrome, encephalitis, cortical dysgenesis, perinatal encephalopathy, and tuberous sclerosis complex. Best responses were seen in patients suffering from primary generalized epilepsy with tonic-clonic seizures followed by primary generalized epilepsy with atypical absence seizures [14]. Studies have found prominent improvements in seizure control in patients with daily baseline seizures, as in tuberous sclerosis, hypothalamic hamartomas, and Lennox-Gastaut syndrome. Nevertheless, Tanganelli et al. reported that, regardless of the type of seizure, VNS therapy in patients with severe encephalopathy was not effective [35].

Ng and Devinsky suggested that VNS can reduce both seizure frequency and medication usage in patients with refractory idiopathic generalized epilepsy significantly compared to those with refractory partial epilepsy [32]. Moreover, in patients with generalized epilepsies, VNS may be more effective in idiopathic disorders than in symptomatic forms [32].

In Ghaemi et al.'s study, the presence of unilateral interictal epileptiform discharges, cortical dysgenesis, and younger age at implantation could independently lead to more seizure-free days [29]. Englot et al. reported that generalized epilepsy, traumatic epilepsy, and tuberous sclerosis were correlated with significantly higher seizure frequency reductions [36]. Patients with posttraumatic epilepsy showed reduction rates of 50% and 73% in 3- and 24-month follow-up sessions, respectively, while in nontraumatic epilepsies, these rates were 46% and 57%, respectively.

Dede et al. reported more favorable VNS outcomes in motor seizure and startle epilepsy [25]. In patients with a history of febrile seizures, central nervous system infection, or brain injury, the reductions in seizure frequency had no remarkable differences [18].

Mental retardation remains a matter of debate as some series show that patients with mental retardation respond favorably to VNS therapy. Arcos et al. confirmed that VNS outcome was correlated with severity of the mental retardation. The level of intellectual disabilities correlated negatively with positive clinical outcomes [18]. Other studies could not detect similar findings.

Comparing the lesional nature and nonlesional nature of seizures, Englot et al. reported that, in epilepsies with lesional etiology, particularly tuberous sclerosis, greatest clinical benefit from VNS is achieved [2]. Colicchio et al. claimed that the lesional etiology (particularly postischemic and tuberous sclerosis) of seizures is associated with the highest percentage of responder to VNS among patients [26]. Further analysis had shown that, in the subgroup of epileptic patients with lesional etiology, a preimplantation epilepsy duration of less than 15 years and an age of less than 18 years at implantation reflect the highest chance of benefiting from VNS.

### 3.5. Duration of Epilepsy

Long duration of epilepsy can cause permanent damage to the central venous system, which can influence the patients' response to VNS. Colicchio et al. have reported that there is a strong link between increases in duration of epilepsy and reduction of clinical response to VNS. They have claimed that patients with more than 21 years of clinical history show worse clinical outcomes with 13% reduction in frequency after one year and 10% after three years [11]. Therefore, seizure reduction rate tends to be inversely correlated with the duration of epilepsy [11]. However, they have claimed that age itself can be a confounding factor in this conclusion and further regression analysis must be performed to determine the effect of these factors on VNS outcomes separately. Englot et al. found that clinical outcome in patients with shorter duration of epilepsy is considerably better than that in those with a seizure history of more than 10 years [1]. Among children, in younger ones with a short duration of epilepsy, VNS therapy is highly efficacious in treatment of seizures [30]. Renfroe and Wheless revealed that patients with seizures of less than 5 years before VNS implantation responded better to treatment compared to patients with longer history of epilepsy [37]. In contrast to previously mentioned studies, Labar has reported that prolonged duration of epilepsy has been associated with more favorable VNS outcomes. Clinical justification of this finding is difficult [31]. Consistant with the work of Labar, Chrastina et al. confirmed that greater duration of epilepsy was not a negative predictor of VNS efficacy [23]. Another study also claimed that the duration of epilepsy before VNS implantation did not have a remarkable difference between the responder and nonresponder groups [31]. Chrastina et al. denied any relationship between age or epilepsy duration and >50% and >90% seizure frequency reduction rates at 1-year and last follow-up sessions [23]. Although there are some contradictions in the literature, higher duration of epilepsy seems to be associated with worse clinical responses to VNS.

### 3.6. Age at Epilepsy Onset

The impact of age at epilepsy onset on VNS outcomes has also been evaluated in very few articles. Serdaroglu A et al. have reported that parameters including age at VNS implantation, duration of epilepsy, and seizure type were not found to be considerably associated with clinical effectiveness of VNS. The only parameter which was significantly correlated with clinical response was age at epilepsy onset. Patients with early seizure onset show poor outcomes following VNS. [7]. In contrast, Colicchio et al. ruled out age at epilepsy onset as a confounding factor affecting lesional etiology as an independent prognostic factor for prediction of VNS outcomes in epilepsy [26]. Englot et al. also claimed that age of epilepsy onset of more than 12 years is associated with significantly higher degrees of seizure freedom [2].

### 3.7. Frequency of Attacks

The impact of frequency of seizure attacks on VNS outcomes in patients has not been extensively studied in the available literature. One study has reported that patients with a low frequency of seizures respond faster to VNS therapy [18]. Tanganelli et al. reported that, regardless of the type of seizure, VNS in patients with a very high seizure frequency was not effective [35].

### 3.8. Antiepileptic Drugs and VNS

Although the interference of antiepileptic drugs and VNS outcomes is theoretically expectable, there is scarce data regarding this issue in the current literature. Welch et al. [20] reported that the number of antiepileptic agents at 1 year after VNS was not significantly different from that before VNS intervention. García-Pallero et al. evaluated 85 patients who were VNS candidates. Patients were categorized into two groups: a group of those who changed their antiepileptic drugs after VNS implantations and another group of those who did not change their medications. The results showed that, after 18 months, 54.1% of patients had >50% seizure frequency reduction. These figures in change-drug and unchanged-drug groups were 63% and 45.2%, respectively. Statistical analysis revealed no significant difference among these groups [19]. A study by Chrastina et al. showed that, in the subgroup of patients with late VNS response, there was a 54.5% drug dose increase and 27.2% drug change in the year prior to VNS response [21]. Another study by Arcand et al. also reported that medication alterations (dose or type) after VNS were 57%, 33%, 59%, and 81% at 6, 12, 24, and 36 months, respectively. The percentages of VNS responders were 43%, 48%, 41%, and 50% at 6, 12, 24, and 36 months, respectively [24]. As is seen, these rates do not match. These results can be explained just similar to the findings of Chrastina et al. Thus, there is controversy going on regarding the association of treatment changes and optimized function of VNS.

### 3.9. VNS Parameters

Optimal parameter settings for VNS therapy are not yet well defined [14]. Stimulation parameters consist of pulse amplitude, pulse duration, frequency, duration of “on” and “off” times, and ratio of “on” to “off” times. Theoretically, alterations in each parameter can cause changes in the responses produced by patients.

Labar reported that there was no difference in response to VNS between patients who remained on standard cycling and patients who switched from standard cycling to rapid cycling [31]. Gurbani et al. demonstrated a greater reduction in seizure frequency in patients with rapid cycle compared to standard cycle. Pediatric population shows a better response to rapid cycles in comparison to adults [14]. To overcome these discrepancies found in the literature, two meta-analysis reviews were performed. The review of Chambers and Bowen [38] showed that high-stimulation modes had significantly higher efficacy for reduction of seizures compared to low-stimulation modes in adults. There was no significant difference between these two paradigms in the pediatric population. Another meta-analysis by Panebianco et al. also showed that >50% reduction in seizure frequency is more probably seen with therapeutic mode compared to low-stimulation settings [39]. More research with larger population is recommended for further study of these parameters.

### 3.10. EEG Findings

EEG findings reflect the status of epilepsy in patients. Dede Ho et al. have suggested that VNS would be more beneficial if EEGs demonstrate bilateral and multifocal discharges [25]. Another study concluded that, in patients with bilateral discharge compared to unilateral discharge, greater responsiveness was observed [13]. In contrast, Tecoma and Iragui [33] found that patients with independent epileptic foci in both hemispheres may exhibit worse responses to VNS. Another study also emphasized that absence of bilateral interictal epileptiform discharges on the EEG could result in a better outcome [18]. This study has also analyzed the discharges from temporal lobe with or without contribution of other brain regions in forming seizures. They concluded that presence of temporal lobe discharges is associated with significantly better VNS responses.

Compared to patients without discharges from temporal lobe, this study suggests temporal lobe discharge as an early indicator of response [18]. Complete normalization of the EEG abnormalities in all children who became seizure-free was reported in Serdaroglu et al.'s investigation [7]. A study revealed conflicting results regarding EEG findings in patients after VNS. In that study, 10 out of 17 patients showed improvements at 3 and 6 months after VNS on EEG but four patients showed more abnormalities on EEG 6 months after VNS compared to 3 months after VNS. [22] Another study revealed that alpha, theta, delta, and beta bands had significantly higher pdBSI values in nonresponders than in responders. It is reported that pdBSI values of alpha and theta are significant indices in predicting responders and nonresponders to VNS [28]. Further research for clarification of precise and exact effects of VNS on EEG is warranted.

### 3.11. MRI Findings

Findings of MRI can show correlations with VNS outcome measures. According to Janszky et al.'s study, malformation of cortical development on MRI and the absence of bilateral spike focus were correlated with satisfactory VNS outcomes [13]. However, logistic regression analysis showed that only the absence of bilateral interictal epileptiform discharges was independently associated with the efficacy of VNS and the presence of cortical malformations was not ruled out [13]. Arcos et al. [18] reported that, 6 months after VNS, there was no significant difference regarding VNS outcomes between patients with abnormal and normal MRI but, after 12 months, abnormal-MRI groups showed significantly higher responses to VNS compared to normal-MRI patients (82.4% versus 45%). Similarly, Arya et al. [17] revealed that response rates in abnormal-MRI and normal- MRI patients have been 80.8% and 52.9%, respectively. MRI in post-VNS epileptic patients also needs further studies to be more elucidated.

### 3.12. Biomarkers

Preliminary reports have mentioned that elevations in extracellular norepinephrine levels can be the mechanism of VNS efficacy in epilepsy [27]. The source of norepinephrine has been speculated to be locus coeruleus (LC). Elevations in gamma-aminobutyric acid (GABA) signaling and decreases in excitatory glutamate signaling were also reported in LC [40, 41]. It was shown that transient VNS can commence fast and periodic activity of neurons in LC but increases in pulse and intensity of VNS can dramatically increase the neuronal activity in LC. In addition to LC, elevation in CSF GABA after VNS is also reported [42]. Thus, measurement of norepinephrine and GABA in experiments can be considered as a key to prediction of VNS function.

### 3.13. Side Effects of VNS

VNS has been proven to be safe [7, 15, 16] and well tolerated [16] and is usually not associated with the common central nervous system adverse effects [7].

Most patients will face mild and transient stimulation-related side effects including vocal hoarseness (the most frequent adverse effect [18, 25]), sore throat, paresthesia, anorexia, and cough, which occur only during stimulation and resolve over time. Common stimulation-related side effects (e.g., cough, voice alteration, and throat paresthesias) are typically mild and can be reversed by adjusting the stimulation parameters [3]. Neck sensory abnormalities in up to 15%, voice hoarseness in up to 50%, cough in up to 15%, and dyspnea in up to 15% of patients are reported at 12 months but they are reduced to 5%, 20%, 1%, and 3% at five years, respectively [43]. Complications associated with implantation include infection at the incision site (may lead to the removal of the stimulator), lead impedance problem, and transient paralysis of the left vocal cord [7]. A study has demonstrated that adverse effects are fewer in patients below 12 years of age [30]. In another study, adverse events occurred within 48 hours after increasing stimulation parameters and then resolved either spontaneously or via substantially decreasing stimulation parameters [6]. The infectious complications need intravenous antibiotics or removal of stimulator [6]. Cough, paresthesia, pain, dyspnea, headache, infection, and asystole are also occasionally reported [1]. Unilateral vocal cord paralysis, seroma/hematoma requiring aspiration, pneumothorax, hoarseness, dysphagia, superficial, and deep infections were seen in Elliott et al.'s study [12]. A review of 247 VNS implantations showed an overall implantation complication rate of 8.6%. Specifically, the most common complications were as follows: infection (2.6%), postoperative hematoma (1.9%), and vocal cord palsy (1.4%) [44].

### 3.14. VNS versus Other Neurostimulation Therapies

There are other neurostimulation systems available for the treatment of refractory epilepsy. Responsive Neurostimulation (RNS) continuously senses electrographic activity via depth or cortical strip leads implanted based on the patient's seizure foci and it works by detecting seizures at start and then stimulating the seizure focus to prevent the propagation [45]. Randomized controlled trials have demonstrated RNS efficacy in patients with one or two seizure foci [46]. In a study by Wang et al., the authors compared the outcomes of VNS versus RNS. They concluded that there is no statistically significant difference between VNS and RNS in terms of reduced seizure frequency for patients with temporal lobe epilepsy; however, this study suffers from small sample size and different duration of follow-up for VNS and RNS groups [47].

Deep brain stimulation (DBS) of the anterior thalamic nuclei (ATN) is another approved therapy that has shown efficacy in randomized trials. In SANTE trial, the median seizure reduction in the stimulation increased from 21.3% after electrode placement to 40.4% after 90 days [48]. In a long-term follow-up of patients treated with ATN-DBS, median seizure reduction increased from 41% at one-year follow-up to 69% after five years. Additionally, responders increased from 49% to 68% after five years [49]. There is some evidence suggesting that VNS and ATN-DBS share common pathways and the thalamus is consistently involved in VNS therapy. Positron emission tomography (PET) and functional magnetic resonance imaging (fMRI) have shown the effects of VNS on higher brain structures through the vagus nerve [50]. Kulju et al. have reported similarities between the responses to ATN-DBS and VNS. In their study, the authors evaluated eleven patients with previous VNS therapy who underwent ATN-DBS implantation. They reported that the response to ATN-DBS therapy seems to be correlated to the response to previous VNS therapy; if a patient had positive effect of VNS, ATN-DBS effect also showed the same effect. In VNS therapy nonresponder patient, the chances for a stable response to DBS were reduced as well [51].

Outcome data of different neurostimulations show that they are an effective, yet palliative approach. Current evidence does not provide sufficient data regarding the superiority of one neurostimulation over another. More studies are warranted to compare efficacy and safety of available neurostimulations.

## 4. Limitations

Given heterogeneity of patients undergoing VNS implantation and the degree and quality of presurgical workup, currently there is no clear agreement on factors associated with response to VNS.

## 5. Conclusion

It is important to take into consideration the fact that complete seizure freedom is rarely achieved using VNS [15, 36]. Based on the current literature, nonresponder rate ranges between 25% and 65%. Thus, it is essential to select the best candidates for VNS before implantation [25], given the probable adverse effects. Given the complexity and diversity of factors associated with response to VNS, more clinical studies are needed to establish better paradigm for selection of patients for VNS therapy.

## Figures and Tables

**Table 1:** Summary of studies evaluated in the review.

Author/year	Study design	Study population	Diagnosis	Outcomes
Welch et al. (2018) [20]	Retrospective chart review	11 patients	Primary generalized epilepsy	(i) 64% improved seizure frequency (ii) No association between epilepsy classification or age at implantation and VNS outcome
Chrastina et al. (2018) [21]	Retrospective study	74 patients	Drug-resistant epilepsy	(i) No significant predictor of VNS outcome was found
Liu et al. (2018) [22]	Prospective study	17 patients	Drug-resistant epilepsy	(i) 2/17 improved EEGs in 3 months and 10/17 improved EEGs in 6 months (ii) No factor with significant impact on EEG
García-Pallero et al. (2017) [19]	Prospective study	85 patients	Medication-resistant epilepsy	(i) No factor was correlated with VNS outcomes
Chrastina et al. (2017) [23]	Retrospective study	103 adults	Long-lasting epilepsy	(i) Worse VNS outcomes were not correlated with older age or longer epilepsy duration
Arcand et al. (2017) [24]	Retrospective review	30 adult patients (18 females and 12 males, 35.1 ± 13.3 years)	Drug-resistant epilepsy	(i) No significant factor associated with VNS outcomes
Ho et al. (2017) [25]	Retrospective study	17 adult cases (M/F: 12/5)	Drug-resistant epilepsy	(i) Favorable clinical response (58.8%) (ii) 50% well response in structural or metabolic disorder of the brain and 63.6% in the genetic or unknown etiology (iii) EEG lateralization correlated with good VNS outcome (*p*=0.007)
Bodin et al. (2016) [6]	Retrospective chart review	29 children (12 females and 17 males)	Drug-resistant epilepsy	(i) 59% response at 3 months and 66% at 6 months (ii) More effective in nonidiopathic partial epilepsy compared to nonidiopathic and idiopathic generalized epilepsy (iii) No other factor correlated with VNS outcome
Gurbani et al. (2016) [14]	Retrospective chart	35 patients (<12 years of age)	Intractable epilepsy	(i) Clinical response in 68.9% of generalized epilepsy and 50% of focal epilepsy cases (*p* < 0.05)
Serdaroglu et al. (2016) [7]	Retrospective chart review	56 patients (aged 4–17 years at implantation)	Intractable epilepsy	(i) Response rate of 9.8% at the 6th month, 24% at the 2nd year, 46.4% at the 3rd yr, and 54% at the 5th year (ii) Seizure onset correlated with outcome (more favorable with higher ages of onset)
Meng et al. (2015) [16]	Retrospective study	106 patients	Adult and pediatric pharmaco-resistant epilepsy	(i) No factor was associated with VNS outcome
Lagae et al. (2015) [4]	Prospective cohort	70 patients between 19 months and 25 years	Drug-resistant epilepsy (*n* = 16 focal and 54 generalized epilepsies)	(i) 54% clinical response (5.7% seizure-free) (ii) No difference in outcome between focal and generalized epilepsies (iii) Age at implantation significantly associated with outcomes (<5 years with 77% response, 75% of seizure-free cases)
Englot et al. (2015) [2]	Retrospective chart review of patients and literature review	5554 from VNS therapy patient outcome registry + 2869 from literature review	Intractable epilepsy	(i) 49% response in 0 to 4 months after VNS (5.1% seizure-free) (ii) 63% response in 24 to 48 months after VNS (8.2% seizure-free) (iii) Seizure freedom predicted by age of epilepsy onset >12 years and generalized seizure type (iv) Overall response to VNS predicted by nonlesional epilepsy
Arya et al. (2014) [17]	Retrospective chart review	43 patients (23 males and 20 females, 14.1 ± 5.4 years)	Childhood-onset epilepsy	(i) 9.3% seizure-free (ii) Absence of lesion on MRI and duration of epilepsy before implantation were significant predictors of favorable outcome
Arcos et al. (2014) [18]	Retrospective study	40 patients	Refractory epilepsy	(i) Baseline frequency of seizures, temporal lobe discharge on EEG, and MRI lesions were predictors of VNS outcome
Champeaux et al. (2012) [8]	Retrospective review of cases	101 patients (57 males)	Refractory epilepsy	(i) Favorable response rate of 41.6% (ii) No factor affecting VNS outcome
Colicchio et al. (2012) [26]	Prospective study	53 patients (33 males and 20 females)	Drug-resistant epilepsy	(i) 40% response rate (ii) Better response to VNS was associated with lesional etiology (*p*=0.017), tuberous sclerosis (*p*=0.022), and age at implant <18 years (*p*=0.024)
Englot et al. (2011) [1]	Retrospective chart review of case series	(i) 3 months after VNS therapy (*n* = 4483, 238 patients <6 years, 1422 patients 6–18 years, and 2823 patients >18 years) (ii) 6 months after VNS therapy (*n* = 3040, 155 patients <6 years, 937 patients 6–18 years, and 1948 patients >18 years) (iii) 12 months after VNS therapy (*n* = 2698, 111 patients <6 years, 831 patients 6–18 years, and 1756 patients >18 years) (iv) 24 months after VNS therapy (*n* = 1104, 30 patients <6 years, 294 patients 6–18 years, and 780 patients >18 years)	(i) Aura-only (*n* = 76, 55, 49, and 19 for 3, 6, 12, and 24 months after VNS) (ii) Simple partial seizure (*n* = 300, 212, 182, and 86 for 3, 6, 12, and 24 months after VNS) (iii) Complex partial (*n* = 2020, 1376, 1241, and 531 for 3, 6, 12, and 24 months after VNS) (iv) Primary generalized tonic-clonic (*n* = 484, 316, 275, and 124 for 3, 6, 12, and 24 months after VNS) (v) Secondarily generalized tonic-clonic (*n* = 325, 238, 214, and 97 for 3, 6, 12, and 24 months after VNS) (vi) Absence (*n* = 284, 196, 158, and 59 for 3, 6, 12, and 24 months after VNS) (vii) Atonic (*n* = 225, 139, 142, and 45 for 3, 6, 12, and 24 months after VNS) (viii) Other (*n* = 399, 269, 229, and 73 patients for 3, 6, 12, and 24 months after VNS)	(i) Progressive increase of clinical benefit with treatment duration (46% decrease in seizure frequency 3 months after VNS, 56% 1 year after VNS, and 62% 2 years after VNS) (ii) Significant difference in clinical response between age groups (60% reduction in <6 years and 6–18 years groups and 53% > 18 years group, *p*=0.0018) (iii) More favorable response to VNS in shorter durations of epilepsy (iv) Significant difference in clinical response between different seizure types (*p* < 0.001); greatest clinical benefit in simple-partial seizures, the least response in generalized tonic-clonic seizures
Raedt et al. (2011) [27]	Prospective study	Male Wistar rats	Animal model of limbic seizures	(i) Significant association between increased hippocampal noradrenaline concentration and favorable VNS outcomes
De Vos et al. (2011) [28]	Prospective study	19 patients	Medically refractory epilepsy	(i) Higher pdBSI values for delta, alpha, theta, and beta bands of EEG associated with nonresponse compared to response (ii) Theta and alpha bands as significant indices for prediction of clinical response
Elliot et al. (2011) [12]	Retrospective review	436 patients (50.5% females, 1–76 years)	Treatment-resistant epilepsy	(i) Focal EEG findings predicted improved outcomes (*p*=0.004) (ii) A trend for better outcome in patients with exclusively focal seizures (*p*=0.09) (iii) Most significant response in focal or temporal epilepsy
Burakgazi et al. (2011) [5]	Retrospective analysis of case series	46 patients (mean age = 45.35 ± 12.73 years, 50% males)	Medically intractable epilepsy	(i) 41.3% satisfactory outcome (ii) 65% frontal lobe epilepsy and 15% temporal lobe epilepsy had satisfactory outcome (iii) Significant correlation between type of epilepsy and outcome (*p*=0.004)
Colicchio et al. (2010) [11]	Retrospective chart review	135 patients (0.5–6 years, *n* = 18; 7–12 years, *n* = 32; 13–18 years, *n* = 31; and >18 years, *n* = 54)	Drug-resistant epilepsy (Lennox-Gastaut *n* = 18, severe multifocal epilepsy *n* = 33, partial epilepsy *n* = 84)	(i) Significant positive correlation of clinical response and treatment duration (*p*=0.04) (ii) Significant effect of age of implant on outcome (worse outcome in adults compared to children) (iii) Significantly better outcome in severe multifocal epilepsy compared to Lennox-Gastaut and partial epilepsy (*p*=0.03) (iv) Positive impact of shorter epilepsy duration on VNS outcome
Ghaemi et al. (2010) [29]	Retrospective study	144 patients	Refractory epilepsy	(i) 6.9% seizure-free (ii) Younger age at implantation, cortical dysgenesis, and unilateral interictal epileptiform discharge were associated with seizure freedom
Kuba et al. (2008) [15]	Retrospective, multicenter, open-label study	90 patients	Drug-resistant epilepsy	(i) 44.4%, 58.7%, and 64.4% clinical response at 1st, 2nd, and 5th years after VNS (ii) Patients <16 years had lower favorable outcomes following VNS
Rychlicki et al. (2006) [30]	Prospective study	34 children (11.5 years of age)	Partial refractory epilepsy	(i) 39%, 38%, 49%, and 61% seizure reduction at 3, 6, 12, and 24 months after VNS (ii) Partial epilepsy (iii) With or without drop attacks, had better outcomes compared to Lennox-Gastaut syndrome
Janszky et al. (2005) [13]	Retrospective chart	47 patients (22.7 ± 11.6 years)	Treatment-resistant epilepsy	(i) 13% seizure-free after VNS (ii) Significant association between absence of bilateral interictal epileptiform discharges (IED) and presence of malformation of cortical development (MCD) with VNS outcome (iii) A trend to negative association between epilepsy duration and outcome
Labar et al. (2004) [31]	Retrospective study	269 patients	Patients on unchanged antiepileptic drugs	(i) Clinical response of 45% at 3 months and 58% at 6 months (*p* < 0.001), older age, longer epilepsy duration, and syndromes other than Lennox-Gastaut (*p* values of 0.016, 0.033, and 0.003) were associated with VNS outcome
Ng et al. (2004) [32]	Retrospective study	165 patients	Medically refractory epilepsy (138 partial epilepsy (PE); 13 symptomatic generalized epilepsy (SGE) and idiopathic generalized epilepsy (IGE))	(i) Clinical response in 47.1% PE, 46.1% SGE, and 57.1% IGE (ii) Reduced antiepileptic drug regimen in 9.4% PE, 7.7% SGE, and 35.7% IGE
Kawai et al. (2002) [10]	Prospective cohort	13 patients (59% males)	Medically intractable epilepsy	(i) >60% reduction in seizure frequency in 15%, 46%, 54%, and 69% for 1st to 4th year after VNS (ii) VNS outcome correlated positively with seizure severity
Patwardhan et al. (2000) [9]	Prospective cohort	38 children (11 months–16 years)	Medically refractory epilepsy	(i) 68% clinical response (ii) 80% response in atonic seizures, 65% in absence, 48% in complex partial, and 45% in generalized tonic-clonic (iii) Significantly better outcome in patients with onset of epilepsy after 1 year of age (*p* < 0.05)

## Data Availability

The data supporting this review article are from previously reported studies and datasets, which have been cited.
